# Peptide Sodium Channels Modulator Mu-Agatoxin-Aa1a Prevents Ischemia-Reperfusion Injury of Cells

**DOI:** 10.3390/molecules28073174

**Published:** 2023-04-03

**Authors:** Elena Iurova, Evgenii Beloborodov, Eugenia Rastorgueva, Aleksandr Fomin, Yury Saenko

**Affiliations:** 1 Laboratory of Research and Development of Peptide Drugs and Vaccines, S. P. Kapitsa Technological Research Institute, Ulyanovsk State University, Ulyanovsk 432017, Russia; urovaev523@gmail.com (E.I.); beloborodov.evgeniy.a@gmail.com (E.B.); rastorgueva.e.v@yandex.ru (E.R.); mr.fominan@yandex.ru (A.F.); 2 Department of General and Clinical Pharmacology and Microbiology, Faculty of Medicine, Ulyanovsk State University, Ulyanovsk 432017, Russia

**Keywords:** sodium, peptide toxin, ischemia reperfusion injury, apoptosis

## Abstract

Ischemia-reperfusion injury (IRI) is an irreversible functional and structural injury. Restoration of normal oxygen concentration exacerbates the emergence and development of deadly cells. One of the possible moments of reperfusion damage to cells is an increase in the intracellular concentration of sodium ions. In this article, we study the mu-agatoxin-Aa1a, a modulator of sodium channels, on the processes of IRI cells damage. The toxin was synthesized using an automatic peptide synthesizer. Hypoxia was induced by reducing the content of serum and oxygen in the CHO-K1 culture. The influence of the toxin on the level of apoptosis; intracellular concentration of sodium, calcium, and potassium ions; intracellular pH; totality of reactive oxygen species (ROS), nitric oxide (NO), and ATP; and changes in the mitochondrial potential were studied. The experiments performed show that mu-agatoxin-Aa1a effectively prevents IRI of cells. Toxin reduces the level of apoptosis and prevents a decrease in the intracellular concentration of sodium and calcium ions during IRI. Mu-agatoxin-Aa1a contributes to the maintenance of elevated intracellular pH, reduces the intracellular concentration of ROS, and prevents the decrease in intracellular NO concentration and mitochondrial potential under conditions of reoxygenation/reperfusion. An analysis of experimental data shows that the mu-agatoxin-Aa1a peptide has adaptogenic properties. In the future, this peptide can be used to prevent ischemia/reperfusion tissue damage different genesis.

## 1. Introduction

Ischemia-reperfusion injury (IRI) of organs and tissues is an irreversible functional and structural damage resulting from reperfusion after prolonged ischemia and hypoxia. IRI is a direct cause of death and disability in patients after myocardial infarction and cerebrovascular accidents and after organ and tissue transplants [1,2,3].

In case of IRI, several pathological processes associated with impaired cellular function occur, which lead to cell damage and their death. The restoration of the normal oxygen concentration in hypoxic cells and tissues occurs paradoxically, exacerbates damage, and also causes cell death. During hypoxia, anaerobic metabolism becomes the main one, which leads to a significant decrease in the intracellular ATP concentration and cytoplasmic pH. Compensation for the decrease in pH occurs due to the activation of Na^+^/H^+^ channels, which leads to the removal of hydrogen ions from the cytoplasm, with a simultaneous influx of sodium ions [4]. Simultaneously with the entry of sodium ions into the cell, the osmotic balance is upset, and the cell swells. The mechanisms of transport of potassium ions from the cell to the intercellular space are triggered to prevent further swelling of the cell [5]. Sodium ions are also replaced for Ca^2+^ ions, which leads to an increase in the intracellular concentration of these ions. During reperfusion, the intracellular concentration of Ca^2+^ ions rises even more, which is associated with the restoration of oxidative phosphorylation processes and an increase in the activity of the Krebs cycle [6]. Elevated intracellular calcium concentration activates mitochondrial Ca^2+^-dependent proteases such as calpain, which in turn converts the cellular enzyme xanthine dehydrogenase to xanthine oxidase. During hypoxia, ATP degradation leads to the formation of hypoxanthine. With reperfusion, when oxygen supply is restored, xanthine oxidase catalyzes the decomposition of hypoxanthine to uric acid, superoxide anion radical, and hydrogen peroxide [7]. This process leads to intracellular oxidative stress.

Evidence is accumulated that sodium overload is caused by increased sodium flux into the intracellular space from the extracellular space under conditions of ischemia and hypoxia and may play an important role in the genesis of IRI [8]. It has been shown that inhibition of sodium entry pathways into cardiomyocytes by pharmacological agents reduces sodium overload during ischemia and thus leads to better functional recovery after reperfusion [9,10].

There is a group of peptides in nature that are selective blockers of Na^+^ channels. They are found in the toxins of arachnids. These peptides contain cysteine-rich peptides and disulfide bridges, which form a unique cystine pseudoknot that provides stability under different conditions [11]. These proteins bind highly selectively to voltage-gated ion channels of the cell and inhibit them, thereby preventing the entry of ions into the cell from the intercellular space or from the endoplasmic reticulum.

Thus, the search for new targeted peptides capable of inhibiting sodium channels is an important problem of contemporary pharmacology. In this paper, the focus of the study was on the effect of the toxin mu-agatoxin-Aa1a, a modulator of sodium channels, on the processes of ischemia-reperfusion cell injury.

## 2. Results

### 2.1. Synthesis Results and Formation of the Secondary Structure of the Toxin

**Figure 1:**
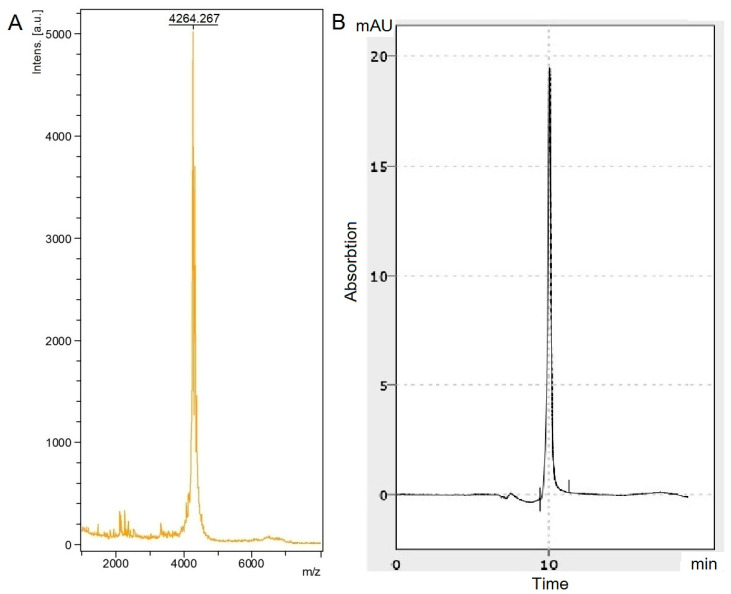
Mass spectrometry (**A**) and chromatogram (**B**) of mu-agatoxin-Aa1a toxin.

The toxin was successfully synthesized through solid phase synthesis. Figure 1 shows the mass spectrogram and chromatogram of the mu-agatoxin-Aa1a peptide toxin after synthesis and purification. The molecular mass of the crude peptide was 4272 Da, but due to the formation of four disulfide bridges, the mass changed to 4264 Da (Figure 1A). The purity of the peptide was 97.6% (Figure 1B).

### 2.2. The Effect of the Toxin on Apoptosis, Necrosis, Cell Area, and Cell Index during Oxygen–Glucose Deprivation/Reoxygenation-Reperfusion

**Figure 2:**
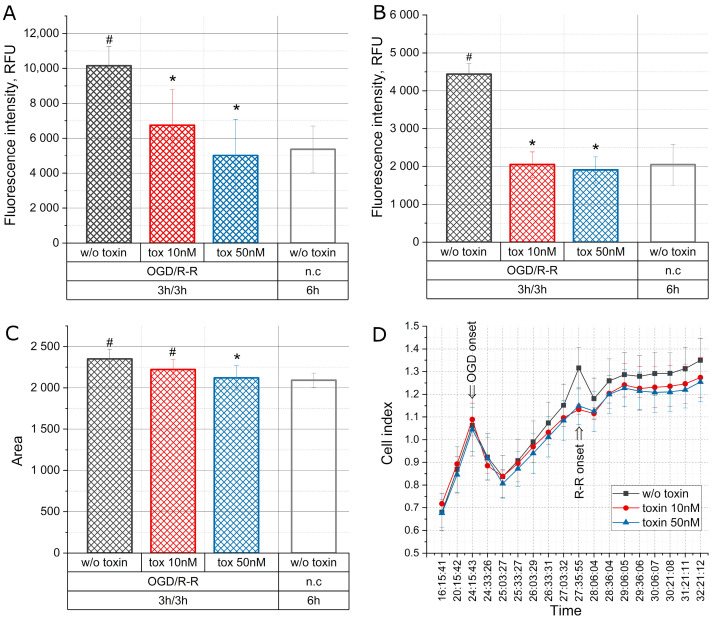
Change in the level of apoptosis (**A**), necrosis (**B**), cell area (**C**), and cell index (**D**) in the CHO-K1 culture with the toxin effect at concentrations of 10 nM and 50 nM under conditions of OGD/R-R (OGD—oxygen–glucose deprivation, R-R—reoxygenation-reperfusion; n.c.—normal condition); RFU—relative fluorescence units); *—*p* < 0.01 when compared with the “OGD w/o toxin” group, #—*p* < 0.01 when compared with the “n.c.” group.

Figure 2 shows the change in the levels of apoptosis and necrosis, the change in the cell area, as well as the change in the cell index in the CHO-K1 culture when simulating the conditions of deprivation of FBS (1%) and glucose (1 g/L) with a reduced oxygen level (1%) (OGD—oxygen–glucose deprivation), followed by 3-h cultivation in a medium containing 10% FBS, 3.151 g/L glucose in 18.6% O_2_ (R-R—reoxygenation-reperfusion) with the toxin at concentrations of 10 nM and 50 nM and in normal conditions.

Figure 2A manifests a two-fold increase in the level of apoptosis during 3 h OGD, followed by 3 h reoxygenation-reperfusion. However, during reoxygenation-reperfusion in the presence of the toxin at concentrations of 10 nM and 50 nM, the level of apoptosis decreased and reached the normal level.

An increase in the level of necrosis was observed under OGD and subsequent reoxygenation in a normal medium which is illustrated in Figure 2B. In the presence of the toxin, the level of necrosis is significantly reduced and remained at the level of normal conditions already at the toxin concentration of 10 nM.

Figure 2C shows the change in the size (area) of cells during the simulation of OGD/R-R conditions. An increase in the cell area relative to normal conditions was observed during the 3 h OGD with subsequent reoxygenation-reperfusion. However, the addition of the toxin to the reoxygenation-reperfusion medium at a concentration of 10 nM did not cause any change in the cell area, and, at a concentration of 50 nM, there was a decrease in the cell area to the value typical for normal conditions.

The analysis of the cell index that characterizes cell adhesion and reflects the response of cells to external impacts demonstrated that, during the first 30 min, there was a drop in the index with OGD (Figure 2D). After that the index increased for 3 h in all the groups. Upon subsequent incubation of the culture under condition of reoxygenation and reperfusion, the index fell again during the first 30 min, but it is noteworthy that in the control group the fall was more rapid than in the groups with the toxin. After that, the index in all the groups continued to increase in the same way.

### 2.3. The Effect of the Toxin on the Concentration of Sodium, Calcium, and Potassium Ions and pH during Oxygen–Glucose Deprivation/Reoxygenation-Reperfusion

**Figure 3:**
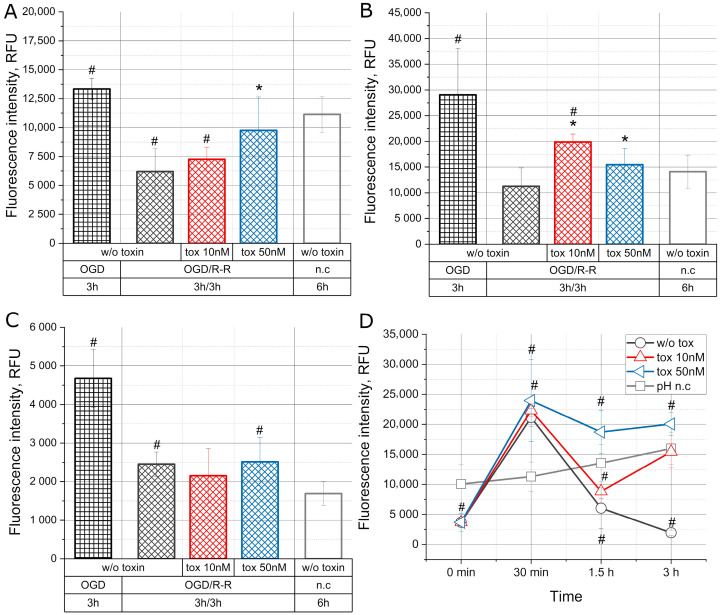
Change in the concentration of sodium (**A**), calcium (**B**), and potassium (**C**) ions and pH (**D**) in the CHO-K1 culture with the toxin effect at concentrations of 10 nM and 50 nM under conditions of OGD/R-R (OGD—oxygen–glucose deprivation, R-R—reoxygenation-reperfusion; n.c.—normal condition); RFU—relative fluorescence units); *—*p* < 0.01 when compared with the “OGD w/o toxin” group, #—*p* < 0.01 when compared with the “n.c.” group.

Figure 3 shows the change in the concentrations of sodium, calcium, and potassium ions and pH when simulating the conditions of OGD/R-R and with the toxin at concentrations of 10 nM and 50 nM, as well as immediately after OGD and under normal conditions.

An increase in the concentration of sodium ions is shown in Figure 3A in the group where the cell culture was incubated for 3 h in OGD. At the same time, after reoxygenation-reperfusion the concentration of ions decreased by about 60%, but the presence of the toxin in the medium at a concentration of 50 nM caused a decrease in the concentration of sodium ions by about 30%, resulting in the indicator dropped to the concentration under normal conditions.

Figure 3B demonstrates that OGD led to a substantially increase in the concentration of calcium ions. Subsequent reoxygenation-reperfusion led to a three times decrease in the concentration to the normal level. However, during co-incubation with the toxin at a concentration of 10 nM, this decrease was moderate, and at a concentration of 50 nM was more rapid.

Figure 3C shows the change in the concentration of potassium ions. One can see that with OGD, there was significant increase in potassium concentration. At the same time, the subsequent 3 h incubation in reoxygenation-reperfusion condition led to a decrease in potassium close to the normal level. The presence of the toxin at concentrations of 10 nM and 50 nM did not have an additional effect on the change in potassium concentration.

In Figure 3D we can see that incubation of cells under conditions of OGD led to acidification of the intracellular milieu, with subsequent alkalization during the first 30 min from the start of reoxygenation in the normal nutrient medium, as a result of which the pH level doubled. Then, during the next 1 h, pH again fell below normal (in the control group by about 70% and in the 10 nM toxin group by about 60%), except for the 50 nM toxin group, where pH remained at the same level. In the next 1.5 h, there was a further drop in pH under control conditions by an additional 20% and an increase in the presence of the toxin at a concentration of 10 nM. At a toxin concentration of 50 nM, the pH remained unchanged for the remainder of the time.

### 2.4. Effect of the Toxin on the Concentration of Reactive Oxygen Species (ROS), Nitric Oxide (NO), ATP, and Mitochondrial Membrane Potential during Oxygen–Glucose Deprivation/Reoxygenation-Reperfusion

**Figure 4:**
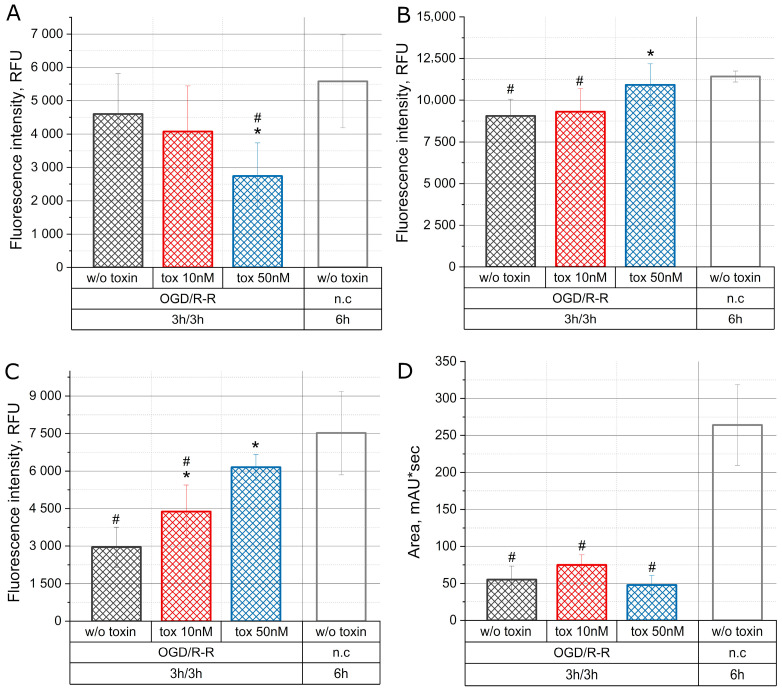
Change in the level of ROS (**A**), NO (**B**), mitochondrial membrane potential (**C**), and concentration of ATP (**D**) in the CHO-K1 culture with the toxin effect at concentrations of 10 nM and 50 nM under conditions of OGD/R-R (OGD—oxygen–glucose deprivation, R-R—reoxygenation-reperfusion; n.c.—normal condition); RFU—relative fluorescence units); *—*p* < 0.01 when compared with the “OGD w/o toxin” group, #—*p* < 0.01 when compared with the “n.c.” group.

Figure 4 demonstrates the change in the concentrations of ROS, NO, ATP and mitochondrial membrane potential in CHO-K1 culture under simulated conditions of OGD/R-R with the toxin at concentrations of 10 nM and 50 nM, as well as under normal conditions.

Figure 4A shows the change in the concentration of reactive oxygen species. When the cell culture was incubated for 3 h under conditions of OGD, followed by a 3 h incubation in normal medium, no changes in the concentration of reactive oxygen species took place. Co-incubation with the toxin at a concentration of 50 nM showed an additional significant decrease in ROS.

Figure 4B demonstrated the change in the NO concentration. One can see that during OGD, followed by a 3 h incubation in a normal nutrient medium at 18.6% O_2_, the concentration of NO decreased by 20% below the normal level. During reoxygenation-reperfusion in the presence of the toxin at a concentration of 10 nM, no changes were observed. However, during reoxygenation-reperfusion in the presence of a toxin at a concentration of 50 nM, the NO concentration remained at the level of normal conditions.

In Figure 4C we can see that a 3 h culture incubation under OGD with subsequent reoxygenation in a nutrient medium during 3 h there was a decrease in the mitochondrial membrane potential by about 60%. The addition of the toxin showed a dose-dependent effect and caused normalization of the mitochondrial potential. Thus, during reoxygenation-reperfusion in the presence of the toxin at a concentration of 10 nM the mitochondrial potential decreased by about 40%, and at a concentration of 50 nM a decrease was not observed, the level of the mitochondrial membrane potential remained within the normal range. The concentration of ATP during reoxygenation-reperfusion reduced by about 80% and the toxin did not affect this parameter (Figure 4D).

## 3. Discussion

Ischemia-reperfusion injury is defined as a paradoxical exacerbation of cell dysfunction and death following restoration of blood flow in previously ischemic tissues. Restoration of blood flow is necessary to save ischemic tissues. However, the reperfusion itself paradoxically causes further damage, threatening the function and viability of the organ. Reperfusion injury is a multifactorial process leading to extensive tissue destruction [12]. Although the main tissues and cells affected by IRI are tissues of the cardiovascular and nervous systems, cells of epithelial origin, such as renal tubular epithelium and lung epithelial cells, are also highly susceptible to IRI [3,13]. In our study, we used the CHO-K1 cell culture, which refers to epithelial cells that endogenously express voltage-gated sodium channels which can serve as a basis for studying the mechanisms of prevention of IRI in tissues and organs [14].

In recent years, an increasing number of studies have been devoted to the study of the effect of ischemic postconditioning on the prevention of IRI, which seems to be a promising approach [15]. Ischemic postconditioning is usually defined as a rapid sequential intermittent interruption of blood flow applied in the early stages of reperfusion. This method is based on the concept that gradual reperfusion of previously ischemic tissue, interrupted by short episodes of ischemia, can provide favorable results [16,17]. Postconditioning leads to a significant reduction in both the severity of systemic inflammatory reactions and the degree of distant damage to the lungs and kidneys [17].

Our study is based on the use of the mu-agatoxin-Aa1a peptide, a sodium channel modulator. Previously, in our works, we have already demonstrated the possibility of blocking apoptosis using the mu-agatoxin-Aa1a peptide [18]. A specific feature of this toxin is that it does not block the ion channel, but instead it seems to freeze it in its current state. Our approach is analogous to the ischemic postconditioning technique, and it is based on the gradual adaptation of ischemic cells to normal conditions. In our experiments, we have used a model based not only on hypoxia, but also on the restriction of cell access to nutrients. This approach, in our opinion, most fully simulates the state of ischemia/reperfusion in vivo.

In our experiments, mu-agatoxin-Aa1a has reduced the level of apoptosis and necrosis in CHO-K1 cell culture already at a concentration of 10 nM (Figure 2A,B). The integral indicator of the cell index, which reflects the response of cells to external influences, is also lower in the groups with the addition of the mu-agatoxin-Aa1a toxin (Figure 2D). When cells were placed under OGD conditions, the cell index increased significantly in all the groups, whereas with reoxygenation/reperfusion, the cell index was higher in the group without toxins.

One of the consequences of IRI is the disruption of the transmembrane movement of ions in cells. It has been experimentally demonstrated that initial hypoxic conditions reduce the current through sodium channels in rat cardiomyocytes, which further decreases during reoxygenation after injury [19,20]. In our experiments, the sodium concentration in CHO-K1 cells slightly increase under hypoxic-deprivation conditions. After 3 h of the onset of reoxygenation/reperfusion conditions, the intracellular concentration of sodium ions decrease by more than 2 times in the group without the toxin (Figure 3A).

The duration of activation and inactivation of sodium channels also correlates with the duration of hypoxic exposure [21,22]. Sodium is the most abundant ion outside the cell, and sodium enters the cell through various electrically neutral cotransporters and exchangers, including the Na^+^/Cl^−^ cotransporter, the Na^+^/K^+^/2Cl^−^ cotransporter, and the Na^+^/H^+^ exchanger in combination with the Cl^−^/HCO_3_^−^ exchanger [23]. It has been shown in [24] that activation of voltage-sensitive sodium channels during oxygen starvation leads to apoptotic caspase-3-dependent neuronal death, while inhibition of sodium channels by tetrodotoxin attenuates caspase-3 activation and apoptosis. Saxitoxin, a sodium channel blocker, prevents anti-Fas-induced apoptosis in Jurkat T cells by preventing sodium influx [25]. Unlike sodium channel blockers, our toxin is a modulator and prevents changes in the current of ions through Na^+^ channels, which is confirmed by the data obtained. As it can be seen from Figure 3a, the presence of the toxin prevents the activation of sodium channels, which is expressed in a less sharp decrease in the intracellular sodium concentration, depending on the dose of the toxin. At a toxin concentration of 50 nM, the intracellular concentration of sodium ions differs slightly from the control group. The toxin also inhibits the cell area growth (Figure 1C), which we also attribute to low sodium channel activity, and increases the intracellular sodium concentration in the presence of the toxin (Figure 3A).

In addition to sodium channels, calcium channels play a certain role in the development of ischemia-reperfusion cell injury. At present, calcium channel blockers are widely used, but their mechanism of action is only speculative, and it is associated with their action on the receptor part of the channel, which is mediated by the state of the channel itself. As studies have shown, L-type calcium channel blockers are effective if they are used in the first hours after the onset of myocardial infarction [26,27]. In our experiments, the intracellular concentration of calcium ions increased significantly with the onset of OGD conditions.

With reoxygenation-reperfusion, the concentration of calcium ions decreases to values close to the control group. The presence of mu-agatoxin-Aa1a slows down the process of reducing the intracellular concentration of calcium ions, which is consistent with the data on the relationship between the intracellular concentration of sodium and calcium [12,28] (Figure 3B). Mu-agatoxin-Aa1a has no effect on the intracellular potassium ion concentration (Figure 3C).

Thus, in our experiment, we have found that the toxin at concentrations of 10 nM and 50 nM has an ambiguous effect on the concentration of calcium and potassium ions, in contrast to the unidirectional effect on sodium ions. We believe that this is due to the fact that we have used a sodium channel modulator, which should not interact with calcium and potassium channels. The concentrations of calcium and potassium ions, under conditions of oxygen–glucose deprivation/reoxygenation-reperfusion, are influenced by a large number of versatile intracellular metabolic and signaling mechanisms, and therefore their intracellular concentration react ambiguously to mu-agatoxin-Aa1a.

During OGD, a significant decrease in cell pH is observed, which returns to normal after reoxygenation-reperfusion. It is believed that acidosis (pH ≤ 7.0) protects cells from death in ischemia. However, the return from acidotic to normal pH after reperfusion leads to loss of cell viability [29]. Effects that cause faster normalization of pH after reperfusion accelerate cell death, while manipulations that delay the increase in pH prevent loss of cell viability [30]. It is believed that during reperfusion, physiological pH is quickly restored due to lactate leaching and activation of the Na^+^-H^+^ exchanger, as well as the Na^+^/HCO-symporter. This pH shift contributes to the death of cardiomyocytes during lethal reperfusion injury of the myocardium [31]. It has been emphasized that a potential strategy to prevent cell damage during reperfusion may be to slow down the normalization of intracellular pH [32].

In our experiments under conditions of OGD, intracellular pH is lower than in the control group, which corresponds to the previously obtained data. In 30 min after the onset of reoxygenation-reperfusion conditions, pH become higher than in the control group, both in the presence of the toxin and without it. Further, the toxin inhibits the decrease in pH and in 3 h of reoxygenation-reperfusion, pH remains higher than in the control group, while in the toxin-free group, pH decreases to the levels observed during OGD (Figure 3D). The high pH value in the presence of toxin may be related to the activity of the Na^+^/H^+^ exchanger (NHE). During ischemia, oxidative phosphorylation rapidly ceases, and anaerobic glycolysis accelerates. The end products of anaerobic glycolysis are lactate and protons [33]. Intracellular acidosis stimulates acid extrusion mechanisms, including NHE and the Na^+^/HCO_3_-cotransporter, which results in one Na^+^ ion for every H^+^ removed. The resulting increase in Na^+^ is minimized by the activation of Na^+^ channels [34]. In our experiments, the activation of Na^+^ channels is blocked by the toxin mu-agatoxin-Aa1a, which prevents the excess removal of incoming Na^+^. Under these conditions, in our opinion, NHE becomes the main mechanism of removing Na^+^, which can contribute to its activation. This explains the fact that, during reoxygenation-reperfusion and in the presence of toxin, a more efficient removal of H^+^ from the intracellular space occurs, which contributes to an increase in intracellular pH in the presence of toxin compared to both the control group and the group without toxin (Figure 3D). Based on our data, we can conclude that mu-agatoxin-Aa1a contributes to the maintenance of elevated intracellular pH under conditions of reoxygenation-reperfusion.

In our experiments, we had not noticed a significant increase in ROS under reoxygenation-reperfusion conditions in all the groups. However, mu-agatoxin-Aa1a at a concentration of 50 nM reduces the intracellular concentration of ROS (Figure 4A).

Numerous experiments have shown that NO reduces the adverse effect of reoxygenation/reperfusion [35] in mice with nitric oxide synthase 3 (NOS3) knockout, the functional recovery after myocardial ischemia/reperfusion is impaired [36], while an increase in the NO concentration accelerates the functional recovery of cells [37]. In our experiments, mu-agatoxin-Aa1a prevented the decrease in the NO concentration under reoxygenation-reperfusion conditions (Figure 4B), which, in turn, favorably affected cell survival (Figure 2).

Endogenous nitric oxide interacts with mitochondrial respiration, possibly through several steps of electron transfer. A low concentration of the donor of NO SNAP increases the membrane potential of mitochondria due to the activation of mitochondrial ATP-dependent potassium channels. Any increase in the mitochondrial membrane potential will result in a decrease in mitochondrial calcium uptake, and indeed, exogenous NO reduces mitochondrial calcium overload during simulated ischemia [38]. In our experiments, we observe that mu-agatoxin-Aa1a prevents the decrease in the mitochondrial membrane potential (Figure 4C), and probably prevents calcium ions overload of mitochondria. The mu-agatoxin-Aa1a toxin has no significant effect on the intracellular concentration of ATP (Figure 4D).

The experimental data analysis shows that the mu-agatoxin-Aa1a peptide has adaptogenic properties, which is expressed in the mitigation of a sharp change in the physiological state of the cell after the restoration of normal environmental conditions. The effect of the mu-agatoxin-Aa1a peptide on cells is comparable to the method of ischemic postconditioning and, in the future, this peptide can be used to prevent ischemia/reperfusion tissue damage different genesis.

## 4. Materials and Methods

### 4.1. Peptide Synthesis and Quality Control

The toxin mu-agatoxin-Aa1a (ECVPENGHCRDWYDECCEGFYCSCRQPPKCICRNNN) (UniProt ID: T5G1A_AGEAP)—a modulator of sodium ion channels—was used in the experiment. Peptide synthesis was carried out with the ResPep SL automated synthesizer (Intavis, Tübingen, Germany) using solid-phase peptide synthesis and applying the Fmoc-chemistry on TentaGel resin according to the manufacturer’s standard protocol. Folding buffer contained 10 mM reduced glutathione and 1 mM oxidized glutathione in 0.1 M Tris-HCl, pH 8.0 at 4 °C with gentle rocking for 24 h [39].

Peptide analysis was performed using a Shimadzu LC-20AD XR chromatographic system equipped with an SPD-20A spectrophotometric detector. The analysis was carried out by reverse phase chromatography using a Dr. Maisch Luna C18 column according to the standard protocol of gradient elution from 95% A; 5% B; followed by an increase in the concentration of eluent B to 100% for 40 min, where eluent A is deionized water, eluent B is acetonitrile (Cryochrom, Saint Petersburg, Russia). Detection was carried out at a wavelength of 215 nm. Mass spectrometric analysis was performed using the MALDI-TOF MS FLEX series hardware and software system (Bruker Daltonics, Bremen, Germany). Purification was performed using HPLC (NGC Quest ™ 10 chromatography system (Bio-Rad, Hercules, CA, USA) using Bio-Gel P-4 sorbent on an Econo-Column 1 × 30 cm column (Bio-Rad, Hercules, CA, USA).

### 4.2. Cell Culture and Experiment Condition

The Chinese hamster cells (CHO-K1 line) (Russian cell culture collection of Vertebrates, Saint Petersburg, Russia) were used in the study. The culture was selected based on the high degree of homology of sodium channels with subtypes of Na^+^ channels in the brain [15]. The cell line was kept in DMEM (PanEco, Moscow, Russia) supplemented with 10% FBS (Biosera, Cholet, France) and gentamicin at a final concentration of 80 μg/mL at 37 °C with 5% CO_2_ in CO_2_ incubator MCO-5AC (Sanyo, Osaka, Japan). Twenty-four hours before the experiment, cells were seeded in 48-well plates (SPL Life Sciences, Pocheon-si, Korea) at a concentration of 40,000 cells per well.

The ischemia/reperfusion model was reproduced under conditions of 3 h cultivation in DMEM medium with a reduced content of FBS (1%) and glucose (1 g/L) in 1% O_2_ and 5% CO_2_ (oxygen–glucose deprivation) in a CB-53 incubator (Binder, Tuttlingen, Germany) followed by a 3 h incubation in DMEM with 10% FBS and 3.151 g/L glucose with 18.6% O_2_ and 5% CO_2_ (reoxygenation-reperfusion). The toxin at final concentrations of 10 nM and 50 nM was added at the beginning of reoxygenation. For general control of some parameters, the cells were incubated under normal conditions (DMEM 10% FBS and 3.151 g/L glucose with 18.6% O_2_, 5% CO_2_). Before the start of each experiment, the nutrient media were equilibrated under the required conditions for 30 min [40].

### 4.3. Measurement of Changes in Intracellular Processes, Area, and pH

In the course of the experiment, changes in the level of apoptosis, necrosis, the mitochondrial membrane potential, ROS, NO, calcium, and sodium ions were recorded. Yo-Pro 1 PI dye (final concentration 1 μM) [41] was used to measure the level of apoptosis and necrosis, TMRE (0.5 μM) was used for the mitochondrial membrane potential [42], DCFH DA (1 μM) for ROS [42], DAF-FM DA (1 µM) for NO [43], Rhod 2 AM (1 µM) for calcium ions [44], ION NaTRIUM Green 2 AM (1 µM) for sodium ions [45], and ION Potassium Green 2-AM for potassium ions [46]. Dyes were added 3 h after the start of reoxygenation or recovery and/or before it started for apoptosis, necrosis, and calcium and sodium ions. Changes in pH were also recorded during reoxygenation and recovery at 0 min, 30 min, 1.5 h, and 3 h. To this end, the dye BCECF DA (1 μM) was added [47].

All dyes were incubated for 20 min at 37 °C in the dark. After staining, all wells were washed twice with warm PBS. The measurement was carried out using a multi-mode microplate reader CLARIO star Plus (BMG LABTECH, Ortenberg, Germany) in 100 µL of PBS in the matrix scan mode (10 × 10). After the experiment, the cells were washed off the plate and their concentrations were calculated. Primary data processing was carried out in the MARS program (BMG LABTECH, Ortenberg, Germany) with subsequent processing in Excel. Fluorescence intensity was expressed in terms of relative fluorescence units (RFU). All data were recalculated per 100,000 cells.

Data processing to calculate the cell area was carried out in the ImageJ program [48] on the basis of photographs obtained using a ZOE microscope (Bio-Rad, Hercules, CA, USA).

### 4.4. ATP Analysis

For ATP analysis, cells after oxygen–glucose deprivation/reoxygenation-reperfusion conditions were washed off the plate with 0.25% trypsin solution (PanEco, Moscow, Russia) and lysed in a buffer containing 0.02 M glycine, 0.05 M Mg^2+^, 0.004 M EDTA (pH 7.4 at 25 °C), when heated in a water bath (100 °C) for 45 s.

The determination of the relative ATP concentration was carried out by anion ion exchange liquid chromatography using an Agilent PL-SAX 4.6 × 150 mm column (PL1551-3802) with application of a Shimadzu LC-20AD XR chromatographic system equipped with an SPD-20A spectrophotometric detector. The analysis was carried out according to the following protocol: 0–2 min—100% A, 2–10 min—linear gradient 0–100% B, 10–14 min—100% B. Where A—deionized water, B—1 M NaCl. Detection was carried out at a wavelength of 257 nm [49].

### 4.5. Cell Index Analysis

An xCellingence RTCA-S16 cell analyzer (ACEA Biosciences, San Diego, CA, USA) was used to determine the change in the cell index [50]. The cells of a 16-well plate were seeded at a concentration of 10,000 cells per well. The culture was incubated for 24 h under normal conditions at 37 °C and 5% CO_2_ in an MCO-5AC incubator (Sanyo, Japan). The cell index was recorded in real time with an interval of 3 h. Then, the nutrient medium in the wells was replaced with the hypoxia medium, and the device was transferred to an incubator with 1% O_2_ and 5% CO_2_ and continued to record the change in the cell index with an interval of 30 min for 3 h. After that, the medium was changed to the medium for reoxygenation, the device was placed in an incubator with 18.6% O_2_ and 5% CO_2_ at 37 °C and the measurement continued. At the end of the measurement, the data were transferred to Excel and processed.

### 4.6. Statistics

Each experiment was performed three times in three repetitions. The results are presented as mean ± standard deviation (M ± SD). The asymmetry and kurtosis criteria were used to determine the nature of the distribution. To assess the statistical significance of differences (due to a small sample size), the Mann–Whitney test was used; processing was performed in the Origin software (OriginLab, Northampton, MA, USA). Since the toxin exposure data were compared with the control conditions without a toxin, as well as with normal conditions, the Bonferroni test was used to eliminate the effect of multiple comparisons, and differences between groups were considered statistically significant at *p* ≤ 0.01.

## Data Availability

The datasets generated during and/or analyzed during the current study are available from the corresponding author on reasonable request.

## References

[1] Chiong M., Wang Z.V., Pedrozo Z., Cao D.J., Troncoso R., Ibacache M., Criollo A., Nemchenko A., Hill J.A., Lavandero S. (2011). Cardiomyocyte death: Mechanisms and translational implications. Cell Death Dis..

[2] Mao R., Zong N., Hu Y., Chen Y., Xu Y. (2022). Neuronal Death Mechanisms and Therapeutic Strategy in Ischemic Stroke. Neurosci. Bull..

[3] Ponticelli C. (2014). Ischaemia-reperfusion injury: A major protagonist in kidney transplantation. Nephrol. Dial. Transplant..

[4] Sanada S., Komuro I., Kitakaze M. (2011). Pathophysiology of myocardial reperfusion injury: Preconditioning, postconditioning and translational aspects of protective measures. Am. J. Physiol..

[5] Pierce G.N., Czubryt M.P. (1995). The contribution of ionic imbalance to ischemia/reperfusion-induced injury. J. Mol. Cell Cardiol..

[6] Murphy E., Steenbergen C. (2008). Ion transport and energetics during cell death and protection. Physiology.

[7] Granger D.N., Korthuis R.J. (1995). Physiologic mechanisms of postischemic tissue injury. Annu. Rev. Physiol..

[8] Takeo S., Tanonaka K., Hayashi M., Yamamoto K., Liu J.X., Kamiyama T., Yamaguchi N., Miura A., Natsukawa T. (1995). A Possible involvement of sodium channel blockade of class-I-type antiarrhythmic agents in postischemic contractile recovery of isolated, perfused hearts. J. Pharmacol. Exp. Ther..

[9] Karmazyn M. (1991). Amiloride enhances postischemic ventricular recovery: Possible role of Na^+^/H^+^ exchange. Am. J. Physiol..

[10] Van Emous J.G., Nederhoff M.G.J., Ruigrok T.J.C., Van Echteld C.J.A. (1997). The role of the Na^+^ channel in the accumulation of intracellular Na^+^ during myocardial ischemia: Consequences for post-ischemic recovery. J. Mol. Cell Cardiol..

[11] De Souza J.M., Goncalves B.D.C., Gomez M.V., Vieira L.B., Ribeiro F.M., De Souza J.M., Goncalves B.D.C., Gomez M.V., Vieira L.B., Ribeiro F.M. (2018). Animal Toxins as Therapeutic Tools to Treat Neurodegenerative Diseases. Front. Pharmacol..

[12] Wu M.-Y., Yiang G.-T., Liao W.-T., Tsai A.P.Y., Cheng Y.-L., Cheng P.-W., Li C.-Y., Li C.J. (2018). Current Mechanistic Concepts in Ischemia and Reperfusion Injury. Cell Physiol. Biochem..

[13] Saren G., Wong A., Lu Y.-B., Baciu C., Zhou W., Zamel R., Soltanieh S., Sugihara J., Liu M. (2021). Ischemia-Reperfusion Injury in a Simulated Lung Transplant Setting Differentially Regulates Transcriptomic Profiles between Human Lung Endothelial and Epithelial Cells. Cells.

[14] Lalik P.H., Krafte D.S., Volberg W.A., Ciccarelli R.B. (1993). Characterization of endogenous sodium channel gene expressed in Chinese hamster ovary cells. Am. J. Physiol. Cell Physiol..

[15] Kumar K., Singh N., Jaggi A.S., Maslov L. (2021). Clinical Applicability of Conditioning Techniques in Ischemia-Reperfusion Injury: A Review of the Literature. Curr. Cardiol. Rev..

[16] Jivraj N., Liew F., Marber M. (2015). Ischaemic postconditioning: Cardiac protection after the event. Anaesthesia.

[17] Li C.-Y., Ma W., Liu K.-P., Yang J.-W., Wang X.-B., Wu Z., Zhang T., Wang J.-W., Liu W., Liu J. (2021). Advances in intervention methods and brain protection mechanisms of in situ and remote ischemic postconditioning. Metab. Brain Dis..

[18] Iurova E., Beloborodov E., Tazintseva E., Fomin A., Shutov A., Slesarev S., Saenko Y., Saenko Y. (2020). Arthropod toxins inhibiting Ca^2+^ and Na^+^ channels prevent AC-1001 H3 peptide-induced apoptosis. J. Pept. Sci..

[19] Cong T.S., Zhang M.H., He H.Y., Lou J.S. (2014). Effects of taurine-magnesium coordination compound on abnormal sodium channel induced by hypoxia-reoxygenation in rat ventricular myocytes. Chin. Pharmacol. Bull..

[20] Liu X.Y., Ding C., Zhang X. (2005). Effect of ischemic-reperfusion on sodium channel current of cardiomyocytes in rats. Chin. J. Clin. Rehabil..

[21] Fröhlich G.M. (2013). Myocardial reperfusion injury: Looking beyond primary PCI. Eur. Heart J..

[22] Gautier G.P., Roccon AO'Connor S., Ruetten H. (2011). Effects of the novel amiodarone-like compound SAR114646A on cardiac ion channels and ventricular arrhythmias in rats. Naunyn Schmiedebergs Arch. Pharmacol..

[23] Pasantes-Morales H. (2016). Channels and volume changes in the life and death of the cell. Mol. Pharmacol..

[24] Banasiak K.J., Burenkova O., Haddad G.G. (2004). Activation of voltage-sensitive sodium channels during oxygen deprivation leads to apoptotic neuronal death. Neuroscience.

[25] Bortner C.D., Cidlowski J.A. (2003). Uncoupling cell shrinkage from apoptosis reveals that Na^+^ influx is required for volume loss during programmed cell death. J. Biol. Chem..

[26] Wang Q.D., Pernow J., Sjoquist P.O., Ryden L. (2002). Pharmacological possibilities for protection against myocardial reperfusion injury. Cardiovasc. Res..

[27] Temkin L.P. (1989). High-dose monotherapy and combination therapy with calcium channel blockers for angina: A comprehensive review of the literature. Am. J. Med..

[28] Kondratskyi A., Kondratska K., Skryma R., Prevarskaya N. (2015). Ion channels in the regulation of apoptosis. Biochim. Biophys. Acta Biomembr..

[29] Bond J.M., Herman B., Lemasters J.J. (1991). Protection by acidotic pH against anoxia/reoxygenation injury to rat neonatal cardiac myocytes. Biochem. Biophys. Res. Commun..

[30] Inserte J., Barba I., Hernando V., Abellán A., Ruiz-Meana M., Rodriguez-Sinovas A., Garcia-Dorado D. (2008). Effect of acidic reperfusion on prolongation of intracellular acidosis and myocardial salvage. Cardiovasc. Res..

[31] Lemasters J.J., Bond J.M., Chacon E., Harper I.S., Kaplan S.H., Ohata H., Trollinger D.R., Herman B., Cascio W.E. (1996). The pH paradox in ischemia-reperfusion injury to cardiac myocytes. EXS.

[32] Cohen M.V., Yang X.M., Downey J.M. (2007). The pH hypothesis of postconditioning: Staccato reperfusion reintroduces oxygen and perpetuates myocardial acidosis. Circulation.

[33] Neely J.R., Grotyohann L.W. (1984). Role of glycolytic products in damage to ischemic myocardium. Dissociation of adenosine triphosphate levels and recovery of function of reperfused ischemic hearts. Circ. Res..

[34] Cross H.R., Radda G.K., Clarke K. (1995). The role of Na^+^/K^+^ ATPase activity during low flow ischemia in preventing myocardial injury: A 31P, 23Na and 87Rb NMR spectroscopic study. Magn. Reson. Med..

[35] Schulz R., Kelm M., Heusch G. (2004). Nitric oxide in myocardial ischemia/reperfusion injury. Cardiovasc. Res..

[36] Jones S.P., Girod W.G., Palazzo A.J., Granger D.N., Grisham M.B., Jourd’Heuil D., Huang P., Lefer D.J. (1999). Myocardial ischemia-reperfusion injury is exacerbated in absence of endothelial cell nitric oxide synthase. Am. J. Physiol..

[37] Kanno S., Lee P.C., Zhang Y., Ho C., Griffith B.P., Shears L.L., Billiar T.R. (2000). Attenuation of Myocardial Ischemia/Reperfusion Injury by Superinduction of Inducible Nitric Oxide Synthase. Circulation.

[38] Bell R.M., Maddock H.L., Yellon D.M. (2003). The cardioprotective and mitochondrial depolarising properties of exogenous nitric oxide in mouse heart. Cardiovasc. Res..

[39] Moore S.J., Leung C.L., Norton H.K., Cochran J.R. (2013). Engineering agatoxin, a cystine-knot peptide from spider venom, as a molecular probe for in vivo tumor imaging. PLoS ONE.

[40] Wenger R., Kurtcuoglu V., Scholz C., Marti H., Hoogewijs D. (2015). Frequently asked questions in hypoxia research. Hypoxia.

[41] Bolaños J.M.G., Morán M., Da Silva C.M.B., Rodríguez A.M., Dávila M.P., Aparicio I.M., Tapia J.A., Ferrusola C.O., Peña F.J. (2012). Autophagy and apoptosis have a role in the survival or death of stallion spermatozoa during conservation in refrigeration. PLoS ONE.

[42] Saenko Y.V., Glushchenko E.S., Zolotovskii I.O., Sholokhov E., Kurkov A. (2016). Mitochondrial dependent oxidative stress in cell culture induced by laser radiation at 1265 nm. Lasers Med. Sci..

[43] Zeidler D., Zähringer U., Gerber I., Dubery I., Hartung T., Bors W., Hutzler P., Durner J. (2004). Innate immunity in Arabidopsis thaliana: Lipopolysaccharides activate nitric oxide synthase (NOS) and induce defense genes. Proc. Natl. Acad. Sci. USA.

[44] Fonteriz R.I., de la Fuente S., Moreno A., Lobatón C.D., Montero M., Alvarez J. (2010). Monitoring mitochondrial [Ca^2+^] dynamics with rhod-2, ratiometric pericam and aequorin. Cell Calcium.

[45] Tay B., Stewart T.A., Davis F., Deuis J., Vetter I. (2019). Development of a high-throughput fluorescent no-wash sodium influx assay. PLoS ONE.

[46] Camilli G., Bohm M., Piffer A.C., Lavenir R., Williams D.L., Neven B., Grateau G., Georgin-Lavialle S., Quintin J. (2020). β-Glucan–induced reprogramming of human macrophages inhibits NLRP3 inflammasome activation in cryopyrinopathies. J. Clin. Investig..

[47] Alvarez-Leefmans F.J., Herrera-Pérez J.J., Márquez M.S., Blanco V.M. (2006). Simultaneous Measurement of Water Volume and pH in Single Cells Using BCECF and Fluorescence Imaging Microscopy. Biophys. J..

[48] Schneider C., Rasband W., Eliceiri K. (2012). NIH Image to ImageJ: 25 years of image analysis. Nat. Methods.

[49] Liu H., Jiang Y., Luo Y., Jiang W. (2006). A simple and rapid determination of ATP, ADP and AMP concentrations in pericarp tissue of litchi fruit by high performance liquid chromatography. Food Technol. Biotechnol..

[50] Ke N., Wang X., Xu Y.A.A. (2011). The xCELLigence system for real-time and label-free monitoring of cell viability. Methods Mol. Biol..

